# Global genomic pathogen surveillance to inform vaccine strategies: a decade-long expedition in pneumococcal genomics

**DOI:** 10.1186/s13073-021-00901-2

**Published:** 2021-05-17

**Authors:** Stephen D. Bentley, Stephanie W. Lo

**Affiliations:** Parasites and Microbes, Wellcome Sanger Institute, Wellcome Genome Campus, Hinxton, UK

**Keywords:** Pneumococci, Pneumococcal conjugate vaccine, Genomic, Pathogen surveillance

## Abstract

Vaccines are powerful agents in infectious disease prevention but often designed to protect against some strains that are most likely to spread and cause diseases. Most vaccines do not succeed in eradicating the pathogen and thus allow the potential emergence of vaccine evading strains. As with most evolutionary processes, being able to capture all variations across the entire genome gives us the best chance of monitoring and understanding the processes of vaccine evasion. Genomics is being widely adopted as the optimum approach for pathogen surveillance with the potential for early and precise identification of high-risk strains. Given sufficient longitudinal data, genomics also has the potential to forecast the emergence of such strains enabling immediate or pre-emptive intervention. In this review, we consider the strengths and challenges for pathogen genomic surveillance using the experience of the Global Pneumococcal Sequencing (GPS) project as an early example. We highlight the multifaceted nature of genome data and recent advances in genome-based tools to extract useful information relevant to inform vaccine strategies and treatment options. We conclude with future perspectives for genomic pathogen surveillance.

## Background

*Streptococcus pneumoniae* (or pneumococcus) is a common opportunistic pathogen which causes a wide spectrum of diseases. Infections can range from otitis media to severe invasive pneumococcal disease (IPD) including pneumonia, septicaemia and meningitis. Young children in the first few years of life and elderly adults are particularly susceptible to pneumococcal disease. In 2015, pneumococcal infections were estimated to have caused 8.9 million disease cases, including over 317,000 deaths in children under 5 years old. The heaviest disease burden is in low- and middle-income countries (LMICs) [1].

Pneumococcal disease is preventable by vaccination and treatable using antimicrobials. In the early 2000s, pneumococcal conjugate vaccine (PCV) was first rolled out in high-income countries and then gradually in LMICs via The Global Alliance for Vaccines and Immunization (GAVI) [2]. Different from the previous generation of pneumococcal polysaccharide vaccine (PPV), PCV is immunogenic in infants and induces long-term protection by inducing T cell-dependent immune response. The global deployment of PCV has proven to be very effective in reducing pneumococcal disease worldwide. By 2015, deaths of children aged 1–59 months due to pneumococcal disease were estimated to have declined by 51% [1], in comparison to 2000. PCV has also had a positive impact on reducing antimicrobial resistance both through the direct reduction of highly resistant strains targeted by the vaccine and via a secondary effect through a reduction in febrile illnesses that often require antimicrobial use [3].

PCVs trigger an immune response in the host to target the polysaccharide capsule surrounding the pneumococcal cell [4]. To escape immune clearance, the capsule is constantly under diversification, resulting in 100 currently recognised forms or serotypes [5]. Currently, PCVs target up to 13 serotypes which account for most of the disease in infants, especially those associated with antimicrobial resistance. Incomplete vaccine coverage of serotypes allows the pneumococcal population to evolve and evade the vaccine [6]; there have been several reports of increases in disease due to non-vaccine serotypes [6–12]. Higher valency vaccines targeting up to 24 serotypes are under development [13] and should contribute to reduction in disease caused by the emerging serotypes not covered by 13-valent PCV (PCV13) and continued surveillance is necessary to inform future vaccine strategies.

The Global Pneumococcal Sequencing (GPS) project has been providing genomic surveillance since 2011 [14]. Here, we describe the biology of pneumococcal disease, the genomic approach taken and lessons learned to understand vaccine evasion mechanisms and to track vaccine-evading strains, advances in genome-based characterisation and future perspectives for genomic pathogen surveillance.

## The biology of pneumococcal disease

### Colonisation is a prerequisite for disease

Understanding pathogen biology and disease mechanisms is important to guide vaccine strategy. The pneumococcus is a commensal coloniser of the human nasopharynx, with person-to-person transmission necessary to compensate for regular clearance from the niche by host immunity [15] and competition within the nasopharyngeal microbiome [16, 17]. Therefore, variation within the human nasopharyngeal niche is the main driver of evolutionary change in the pneumococcal genome [18, 19]. In parallel, any systemic antimicrobial use, regardless of its target pathogen, is a major driver of selection [20]. Invasive disease is an evolutionary dead end for the pneumococcus as it will lead to either clearance by antimicrobials, clearance by host immunity or death of the host.

**Fig. 1:**
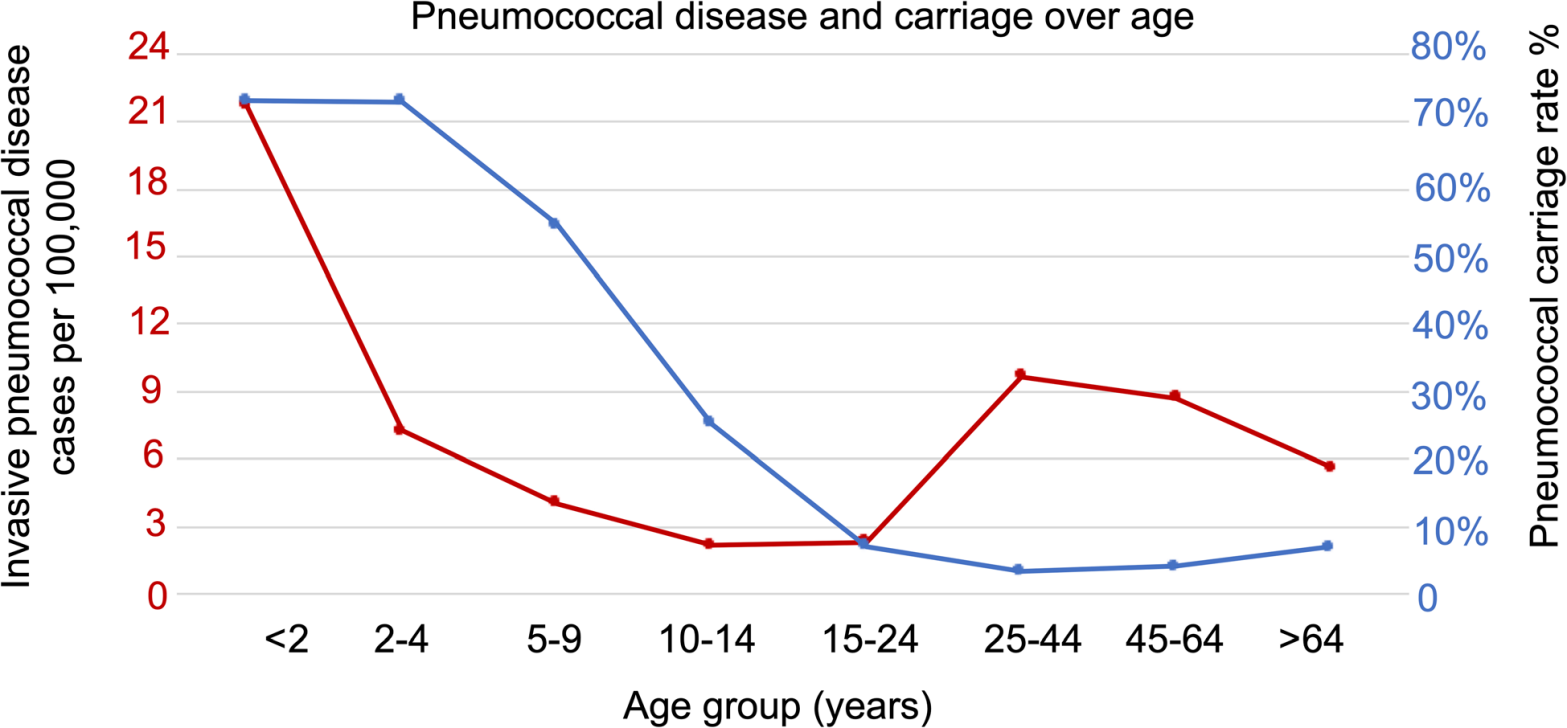
Incidence of invasive pneumococcal disease (red) [29] and carriage (blue) [30] across age groups in South Africa in 2011

Pneumococcal colonisation rates vary with geographical location and age. The colonisation rates in young children are usually lower in high-income countries [21–23] and higher in LMICs [24–26]. Host immunity can also explain the age-related variation, which is highest in infants and declines with maturation of the immune system [27]. It is widely accepted that the primary prerequisite for IPD is prior asymptomatic colonisation with the disease-causing strain, usually in the nasopharyngeal niche [28]. Trends in disease rates roughly follow the age distribution of carriage prevalence though it could be affected by other diseases that can compromise the human immune system (e.g. HIV). In South Africa, a higher incidence rate of IPD in adults > 25 years of age compared to those aged 10–24 years of age (Fig. 1) can be explained by the high burden of HIV in adults > 25 years of age [31]. The incidence of IPD in HIV-infected individuals is estimated to be 43 times higher than HIV-uninfected persons [32]. Interestingly, some serotypes are associated with different age groups [33] and HIV status [34].

### The pneumococcal capsule

The pneumococcal capsule is a layer of cross-linked polysaccharide covering the bacterial cell. One important function of the capsule is to protect pneumococcal cells from phagocytosis [35]; pneumococci without a capsule are usually unable to cause invasive disease, but can cause non-invasive diseases [36]. The capsule is also the basis of the typing scheme which has historically been used to taxonomically separate isolates into groups (serotypes) [37]. Sets of antisera raised against reference “type” strains have been used for over 80 years to serotype isolates, allowing an appreciation of serotype prevalence and relative associations with disease [38]. By serotyping pneumococcal isolates from disease and asymptomatic carriage, substantial variation amongst serotypes in their potential to cause invasive disease was observed [39]. This variation in invasive disease potential is not completely understood but may be linked to the basic biochemical features of the capsule; serotypes with high invasive disease potential tend to have thinner capsules that enhance attachment and direct interaction with epithelial cells [40–42] and are associated with shorter carriage duration periods [43].

The capsule is encoded by a ~ 10–30-kb gene cluster, known as *cps* for capsule polysaccharide synthesis [44]. The composition and sequences of capsular encoding genes vary between serotypes. Analysing these genetic variations paved the way for the development of DNA-based serotyping methods using PCR [45], DNA microarray [46] and whole-genome sequencing (WGS) [47, 48]. These methods show high concordance with the conventional method that is based on reaction to antisera [49–51]. Genotypic methods provide some advantages, including application to culture negative clinical samples [45, 52], detection of multiple co-colonising serotypes [50, 53] and the discovery of novel genetic variations in *cps*, which may indicate new serotypes [5, 54].

**Fig. 2:**
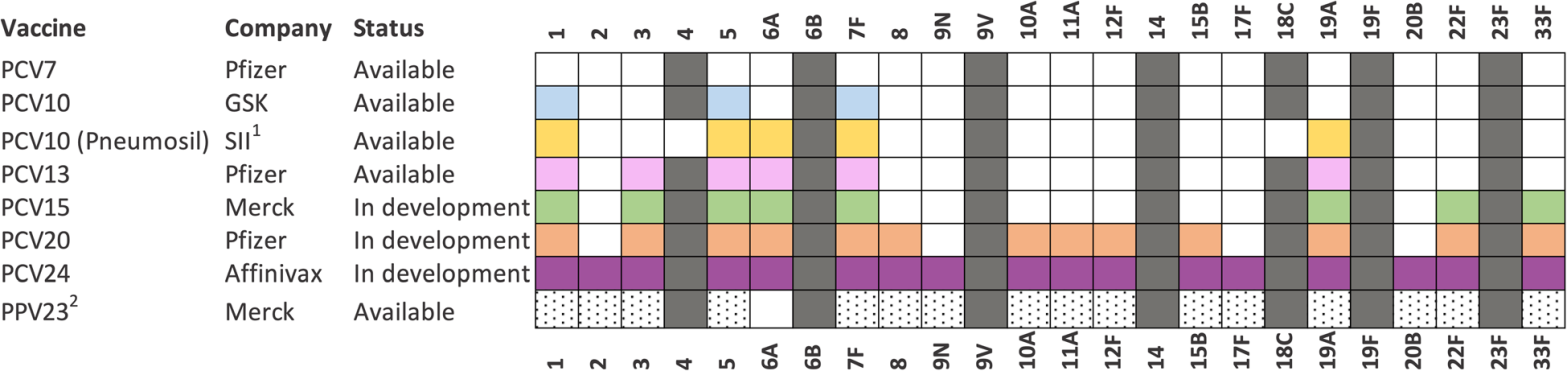
Serotype formulation of pneumococcal vaccines that are currently available and in development. Serotypes included in each vaccine are coloured. Compared to PCV7 serotypes, the additional serotypes in other formulations are coloured in blue (PCV10), yellow (PNEUMOSIL), pink (PCV13), green (PCV15), orange (PCV20), purple (PCV24) and in dotted pattern (PPV23). ^1^SII, Serum Institute of India; ^2^PPV23 is a pneumococcal polysaccharide vaccine which is not immunogenic in children under 2 years of age

Capsular polysaccharide induces a serotype-specific immune response [55] and has been the basis of pneumococcal vaccination since the first clinical use of two different hexavalent PPVs in 1947 [56]. The valency was expanded to 14-valent in 1977 and 23-valent in 1983, offering protection against a wider array of disease-associated serotypes [55, 57]. Unfortunately, PPV induces poor immunogenicity in infants because anti-polysaccharide antibody response is associated with specific splenic B cell subsets that are not fully developed in children under 2 years of age [51]. Additionally, PPV solely elicits a T cell-independent immune response that generates a limited duration of protective antibody level [58, 59]. Considering the disease burden is mainly focused in the first 5 years of life, the above PPV limitations motivated the development of pneumococcal conjugate vaccine (PCV), which would better protect infants. PCV is made by covalently linking capsular polysaccharide to a carrier protein to improve the antibody response and induce long-term protection. PCV is immunogenic in infants and some high-risk patients who do not respond to PPV [60]. The global deployment of PCV since 2000 has been associated with a decreasing pneumococcal disease burden in both children [1] and the indirect protective effect in adults worldwide [61]. Licensed PCVs and those under development, together with 23-valent PPV, are summarised in Fig. 2. Amongst them, the low-cost 10-valent vaccine (PNEUMOSIL) that recently achieved WHO prequalification [62] offers great potential for routine childhood immunisation in LMICs. Although higher-valency PCVs, targeting up to 24 serotypes are under development, the pneumococcal population as a whole has been a moving target for PCVs over the past two decades and the challenge of incomplete coverage of pneumococcal serotypes remains.

## Mechanisms of vaccine evasion

### Recombination and pneumococcal evolution

The pneumococcus is naturally able to uptake naked DNA from the surrounding environment. This characteristic was first demonstrated by Frederick Griffith in 1928 [37] and later used by Avery, MacLeod and McCarty to demonstrate that the ‘transforming principle’ was pure DNA [63]. In the nasopharyngeal niche, the lysis of bacterial cells through normal turnover leads to naked DNA available for uptake which can provide a source of gene variants where different pneumococcal strains are present. Imported DNA can be recombined into the native genome, providing the pneumococcus with a powerful mechanism for rapid evolutionary adaptation [18, 64]. The ability to recombine multi-gene segments of DNA has allowed the import of genetic ‘islands’ from outside of the species and the reassortment of genes within the species, resulting in distinguishable pneumococcal lineages or strains [65]. Recombination enables strains to replace the whole or partial *cps* and thus change serotype [66–68]; this is commonly known as capsular switching. Any switch from serotypes targeted by the vaccine (i.e. vaccine type, VT) to serotypes not targeted by the vaccine (i.e. non-vaccine type, NVT) can contribute to vaccine evasion.

### Vaccine evasion via capsular switching and strain replacement

Multiple capsule switch events have been characterised in a genomic analysis of the globally prevalent PMEN1 strain [67]. Using ancestral phylogenetic reconstruction and recombination analysis of a temporally and geographically broad collection of genomes, it was possible to infer that the strain had likely emerged in Western Europe in the 1970s before spreading globally over following decades. From the serotype 23F ancestor, 10 capsule switch events were detected, some of which were NVT. One notable switch was to serotype 19A which manifested as an emerging cause of NVT disease in the US, after the introduction of PCV7 in 2000 [69]. Ancestral reconstruction showed that the 23F>19A capsule switch had occurred several years before the introduction of PCV7, indicating that the vaccine had created a positive selection for capsule switch variants that were outside of the vaccine coverage.

Pneumococci circulating in any specific geographic region form a multi-strain, multi-serotype population, which is typically dominated by 6–13 strains that together represent > 60% of the population, along with a background of minor strains [51]. PCV have varying effectiveness in removing VTs from the population. The roll-out of PCV tends to have little effect on overall pneumococcal carriage rates, indicating that the NVT portion of the population is able to expand to fill the niche vacated by VTs [70]. After a period of perturbation, the emergent post-vaccine populations appear to have been shaped by the expansion of a combination of capsule switch variants and strains already dominated by NVTs [66, 71]. The relative contribution of these two vaccine evasion mechanisms varies between countries, as does antimicrobial-selective pressure, resulting in variation in post-PCV emerging NVTs. In general, NVTs with high invasive disease potential (e.g. serotype 8, 12F, 24F) are more commonly seen in IPD after PCV13 introduction [6, 8, 9, 71].

## Genomic surveillance to inform global vaccination strategies

### Motivation and scope of the Global Pneumococcal Sequencing (GPS) project

PCV7 was designed to target the serotypes most frequently causing invasive disease in the US. Vaccine coverage was 83% in children aged < 5 years and it was successful in reducing overall IPD by 45% for all age groups over 7 years [72]. In LMICs, PCV was made more affordable through an innovative finance mechanism, the pneumococcal Advance Market Commitment (AMC), initiated by GAVI [2], along with the World Bank and other donors globally in 2009. This mechanism has accelerated the roll-out of PCV to millions of vulnerable children worldwide. However, pneumococcal serotype surveillance indicated that PCV7 would have much lower coverage in many high disease burden LMICs [73, 74]. With this in mind, in 2011 the Bill and Melinda Gates Foundation (in partnership with Emory University, US Centers for Disease Control and Prevention, and the Wellcome Sanger Institute) initiated the GPS project [14] with the primary goal of applying genomics to understand pneumococcal evolution in response to vaccine introduction in LMICs. At that time, GPS was a pioneering project with little precedent to follow, but, 10 years on, lessons have been learned and new directions plotted. The project began with Founding Partners in three African countries (The Gambia, Malawi and South Africa) and the ambition to add partners to achieve wide geographic coverage, prioritising LMICs eligible for GAVI support for PCV rollout. By March 2021, the GPS project sequenced 26,100 pneumococcal genomes representing 57 countries.

Initially, the GPS project prioritised sequencing of isolates from IPD in children under 5 years old, collected pre- and post-PCV introduction. The Founding Partners were from well-resourced institutions, each with a strong track record in pneumococcal surveillance, so were easily able to satisfy the preferred sampling criteria. This was not the case for many other countries and the compromises, such as inclusion of samples from asymptomatic colonisation rather than IPD, were necessary. Allowing such compromises emphasised the importance of careful curation of sample metadata. It was imperative that reliable metadata were collected for every sequenced sample so that specific analytical questions were powered by as many samples as possible; for example, if samples did not have information on whether they were from healthy carriers or IPD, they could not be used in an analysis of genetics associated with virulence. To maximize the utility of the GPS database, no sample was sequenced unless metadata was submitted in advance, thus ensuring that all sequencing effort generated genomic data of enhanced analytical value. The minimal metadata requirement for GPS samples was set simply as ‘date’ and ‘geography’ of isolation, with a range of clinical and microbiological data also typically recorded (see Table 1 for further details). On average, isolates had entries for 37 metadata fields which were linked to the output of genome-derived analyses (e.g. in silico serotype, genotype and antimicrobial resistance determinants). Thus, the GPS provides a rich, public database that has supported a number of data-driven and hypothesis-driven sub-studies with a central theme of pneumococcal disease prevention [75, 76].

**Table 1:** An example of the Global Pneumococcal Sequencing (GPS) project metadata

Categories	Fields	Example
ID	Public name	GPS_ZA_0001
Geographical location	Country	South Africa
	Region	Gauteng
	City	Johannesburg
	Facility where collected	Hospital A
	Submitting institute	NICD
Time	Year	2010
	Month	Aug
Clinical data	Gender	F
	Age (years)	0
	Age (months)	1
	Age (days)	0
	Clinical manifestation	Meningitis
	Source	Cerebrospinal fluid
	HIV status	Negative
	Other underlying conditions	No
Microbiological data	Phenotypic serotype method	Quellung
	Phenotypic serotype	19A
	Multilocus sequence type	ST81
Antimicrobial susceptibility^a^	Method	Broth dilution
	Antimicrobial (e.g. penicillin)	2 mg/L
Selection	Random selection	Y

^a^Antimicrobial susceptibility profile of 17 antimicrobials including penicillin, amoxicillin, cefotaxime, ceftriaxone, cefuroxime, meropenem, chloramphenicol, cotrimoxazole, erythromycin, clindamycin, linezolid, levofloxacin, ciprofloxacin, synercid, tetracycline, rifampin and vancomycin

### Challenges and solutions for genomic surveillance in LMICs

Isolation of *S. pneumoniae* from suspected cases of IPD can be very challenging and may often not be attempted in some countries, necessitating clinical decision making based on other available evidence (e.g. symptoms and prescribing guidelines). Major barriers to pneumococcal isolation from IPD cases include lack of microbiological expertise, lack of correct microbiological reagents (e.g. sheep’s blood rather than human blood) and patient self-administration of antimicrobials prior to presenting to the healthcare provider. Whilst the microbiological barriers can be addressed with training and supply of resources, the issue of uncontrolled antimicrobial access is much more challenging. In countries where culture of IPD isolates is not likely, collecting isolates from the nasopharynx of healthy carriers can be a viable alternative method to evaluate the vaccine impact on pneumococcal population [66] potentially predicting the emerging serotypes/strains post-vaccine using mathematical modelling [77]. However, some serotypes that are frequently found in IPD cases are rarely observed in carriage (e.g. serotype 1), and vice versa [39, 51], so interpretation can be limited.

A fundamental challenge of any global surveillance system, particularly one prioritising LMICs, is variation in local infrastructure and resources, which often also impacts on the level of engagement that an individual project partner is able to commit to. Accordingly, it is important to recognise the motivations and limitations for each partner in order to maximise mutual benefit. Some engagements may be relatively passive, with partners being content to simply contribute culture samples to the project, in the knowledge that analysis of their samples will be reported back to them in the context of regional and global analyses. Others may be more actively involved in developing local genomics capacity and wish to generate and analyse data locally in a way that can be integrated with the global database. Such variation requires flexibility in the global system and failure to provide the necessary flexibility would likely lead to partner disengagement and weakening of the surveillance data captured. In view of such variations, the GPS project devises bespoke support for project partners to cater for different needs in training, data analysis and interpretation.

### Models of sequence data generation: from central to local

Generation of high-quality genome sequence is fundamental to any genomic surveillance system. In the last 2 decades, genome sequencing has progressed from a somewhat cumbersome technology, restricted to a few well-resourced specialist institutions, to become a relatively routine molecular biology tool. In recent years, the sequencing technology companies have developed a greater variety of hardware catering for a variety of uses and budgets. This, coupled with a drive toward genomics as a routine technology for disease surveillance, has led to an expansion in the availability of sequencing hardware in LMICs. In the first phase of GPS (2011–2019) nearly all of the genome sequence data was generated at the Sanger Institute. In the next phase, we have placed a strong emphasis on decentralising data generation in the hope of creating a long-term sustainable genomic surveillance network. It must be acknowledged that the introduction of any new technology takes time, particularly in a resource-limited setting but there are already several high-quality genomics laboratories (e.g. NICD in South Africa [78]) in LMICs and growing networks of national and regional training providers (e.g. MRC unit The Gambia [79] and H3ABionet [80]) so the outlook is positive.

Where data generation is centralised, the movement of samples (bacterial cultures or DNA extracts) presents a significant challenge, often including the need for legal documentation such as material transfer agreements. Assuming decentralised data generation can be achieved, such sample logistic challenges are replaced by data sharing challenges. With the centralised model, outwards data sharing can be relatively straightforward because it emanates from a single uniform data source that has been generated and quality checked. With a decentralised model, there may be variations in data generation so systems need to be developed to enable the data to be harmonised within a unifying data platform. Such systems will need to account for variations in local informatics infrastructure and requirements for legal documentation on data sharing agreements. Data sharing platforms should also be built on open-source software so that the entire stakeholder community can engage in development.

### Database and data sharing

**Fig. 3:**
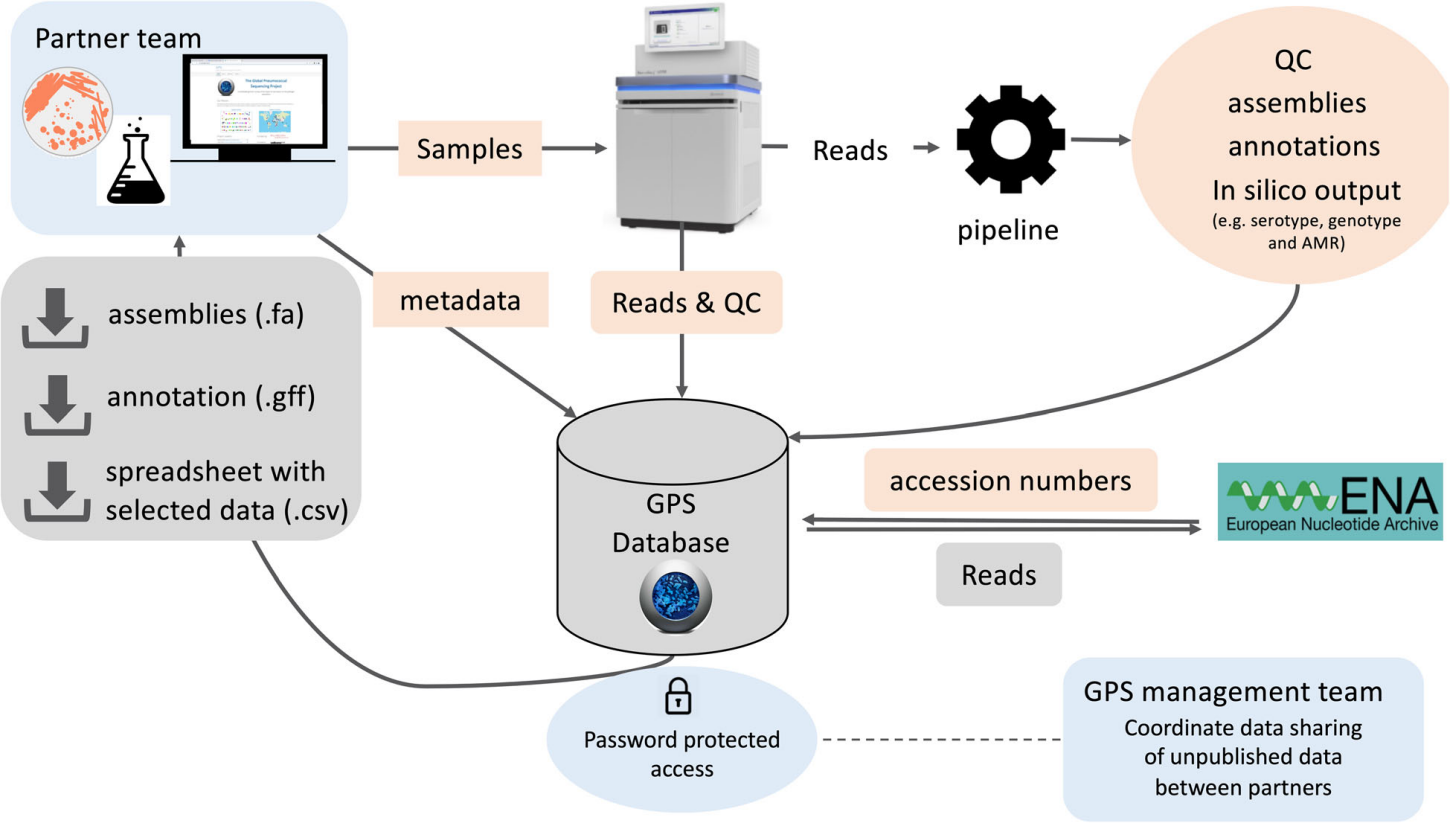
Input and output of the Global Pneumococcal Sequencing (GPS) database. The input is highlighted in light orange whilst output is in grey with downward arrow symbol

The database is an important element of a genomic surveillance system. It serves as a data hub in which a collection of data from multiple sources is organised for users to view, search, download and share. Designing, building and maintaining a database are equally important and all three stages require informatics infrastructure and support. In a surveillance system that involves a network of partners, databases should also be designed to facilitate both individual access to one’s own data and data sharing between partners (Fig. 3).

Data generated from genomic surveillance has great potential value beyond the original purpose so should be publicly accessible. To maximise utility, open data, open software and open access publications are essential and have become strict requirements for many funders [81, 82]. Whilst the availability of open data continues to increase, sharing the benefit arising from the utilisation of these genetic resources in a fair and equitable way is imperative to maintain the virtuous cycle of data production. To this end, the Nagoya Protocol was initiated on 12 October 2014. It provides legal certainty and a transparent benefit-sharing framework for both the genetic resources provider and users [83].

### Data analysis

Translating large amounts of data from a genomic surveillance system into meaningful information to guide public health decisions requires accurate data analysis and interpretation. Over the last decade, a variety of analysis tools have been developed that are robust and generic for application across species. From a pneumococcal genome, we can quickly and reliably extract public health-relevant information, including serotype [47, 48], genotype [84, 85], and antimicrobial resistance profile [86–88]. Such tools are being adapted to be run as applications within websites so formal bioinformatics expertise is not required. For example, Pathogenwatch [89] offers in silico detection and characterisation of genome data for a wide range of microbial pathogens. By simple ‘drag-and-drop’ of sequence data files into a browser window, users can quickly obtain public health-relevant information [90].

Genome data is also powerful in answering key questions, such as the genetic and geographical origin of vaccine evading strains. By calculating substitution rate, we can extrapolate when and where a pneumococcal strain emerged and/or acquired the genetic variation that conferred resistance to the vaccine or antimicrobials [67, 91]. In the first phase of the GPS project 26,100 genomes were sequenced. These data allowed the systematic definition of 621 circulating strains (referred to as Global Pneumococcal Sequence Clusters (GPSCs) and detection of all genomic variations within, including identification of strains containing up to 15 different serotypes [51]. The dataset is dominated by 35 strains (> 100 genomes each) that represent 62% of the dataset; several of these are globally disseminated and associated with multidrug resistance. The GPSC strain definition lays the foundation for understanding pneumococcal population changes after roll-out of PCV. In a GPS study of ~ 3000 pneumococcal isolates from laboratory-based surveillance programmes in six countries collected before and after PCV [71], VTs were replaced by NVTs, as expected [8, 29, 92–94]. Using GPSC, we observed that the expansion of NVTs was mainly mediated by a shift in the balance of serotypes within globally spreading strains, with a smaller impact due to increases of strains that exclusively express non-vaccine serotypes. However, this observation varies amongst countries, as do the prevalent serotypes and GPSCs post-PCV. Such variations can partly be explained by the differences in the pneumococcal population prior to the vaccine roll-out and the variation in antimicrobial selective pressure amongst countries. These data have also enabled the discovery of nine putative novel serotypes [54] and previously unrecognised resistance determinants [95].

### Data visualisation and interpretation

Visualisation of analysed data is a key step for interpretation of large, complex datasets which typically derive from genomic surveillance systems. Visualising genetic relationships between isolates on a phylogenetic tree, together with associated metadata, is a powerful approach. Popular examples of visualisation software include Microreact [96] and NextStrain [97]. The GPS project uses Microreact to make fully analysed datasets easily accessible including snapshots of country-specific [98] and strain-specific studies [99] within project web resources [100, 101]. GPS also uses the Phandango software for visualisation of data specific to gene content variations such as mutation, recombination and pan-genome variations [102, 103].

Interpretation of analysis output requires a certain level of knowledge in bioinformatics and the pathogen studied. In most microbiology laboratories or surveillance networks in LMICs, bioinformatics is a relatively new expertise that requires training and hands-on experience. Together with the sister project JUNO [104], GPS is developing a learning portfolio [105, 106] to suit different partners’ needs informed by a survey that was conducted amongst partners in the GPS and JUNO projects.

## Conclusions and future directions

The GPS project has clearly demonstrated the added value of genomics in pathogen surveillance over the past decade by identifying the emerging serotypes and vaccine-escaping strains, thus providing evidence basis to inform future vaccine strategies. The project also highlighted the data gap and the need to build a more sustainable surveillance system to optimise disease prevention strategies.

### Filling important data gaps in countries with a high burden of disease

In a 2018 study of the global burden of pneumococcal disease, Wahl et al. showed that approximately half of all pneumococcal deaths in 2015 occurred in just four countries: India, Nigeria, Democratic Republic of Congo and Pakistan [1]. However, when that study was published, those four countries represented only 5% of the GPS database. This mismatch was largely due to the difficulty in accessing appropriate samples, with each country having a unique set of economic, technical and political challenges which put them beyond the reach of the initial GPS model. However, there is no lack of capable and motivated stakeholders in those countries and it is hoped that, with a decentralised model and sufficient support for capacity development, those data gaps can be filled. With more representative data, genomic analyses have the potential to give a clear picture of pathogen evolution and risk in the context of regional and global spread.

### Combating multiple pathogens with a generic genomic surveillance system

GPS has already been successful in generating a rich knowledge base for informing future pneumococcal disease control strategy and is making good progress in developing global infrastructure for ongoing genomic surveillance, but there is still much work to be done to achieve a self-sustaining system. Systems for global genomic surveillance of other vaccine-preventable bacterial pathogens are also being established with many solutions likely to be generic across different pathogen species. The most obvious parallels with GPS would be for endemic bacterial pathogens that have similar population structure and incomplete-coverage vaccines. One example is *Neisseria meningitidis* where a variety of vaccine formulations are available but none with complete species coverage. In Africa, where the meningococcal disease burden is highest, widespread use of conjugate vaccine targeting the serogroup A polysaccharide capsule has seen a dramatic reduction in serogroup A disease but also an increase in disease due to other serogroups, most notably serogroup X for which there is currently no licenced vaccine [107]. Meningococcal disease epidemiology in the ‘meningitis belt’ of Africa is characterised by epidemic waves and succession of dominant strains [97]; genomics has great potential for creating a clear understanding of meningococcal population dynamics and creating preparedness for future epidemic waves.

### Enhancing capacity building in LMICs with high disease burden

Genomic surveillance of vaccine-preventable pathogens will only be sustainable through local data generation and analysis which currently places a great emphasis on capacity building in countries with high disease burden. Fortunately, there is a growing wealth of initiatives for training in genomics, including both wet-lab and bioinformatic expertise, with a strong emphasis on the ‘train-the-trainer’ philosophy to ensure sustainability. The supply of sequencing hardware and consumables is improving in many parts of the world that were previously poorly served. Also, advocacy campaigns are raising awareness of the value of genomics with national policy-makers to bring genomics into national disease control strategies. Furthermore, the importance of genomics capacity building in high burden countries is being prioritised by multiple major global health funders. Other fundamental challenges remain. Mechanisms for transfer of funds to the places where they are needed, and protocols for data sharing, need to be made more efficient whilst being sensitive to the needs of the diverse stakeholders. However, by exploiting the universal nature of DNA sequencing and integrating the need to apply genomics to a range of endemic and epidemic pathogens in high burden countries, it should be possible to develop sustainable pathogen genomics surveillance capacity that will have both local and global benefit for infectious disease prevention.

### Optimising vaccine formulation

The WHO lists vaccines “available” for nine bacterial pathogens with differing disease patterns (endemic, epidemic, opportunistic) and differing recommendations for implementation, with some more commonly used in response to outbreaks [108]. In some cases, the vaccine antigen is generally invariant and gives good coverage across the species (e.g. diphtheria, pertussis, tetanus, typhoid). In these cases, low-density genomic surveillance would be valuable in characterising cases of vaccine failure to understand the mechanism of vaccine evasion and to predict whether it is likely to be an emerging threat. In cases where the vaccine antigen is highly variable and the species coverage is partial, it is likely that currently, effective vaccines will need to be periodically reformulated in a manner analogous to the seasonal influenza vaccine. The reformulation cycle may not need to be as rapid as for influenza (annual) and would vary in turnover rate between species. However, having a longitudinal genomic record of pathogen evolution would be enormously valuable in designing new vaccines and potentially forecasting the potential risk/benefit of their use.

Mathematical modelling has provided useful tools for predicting infectious disease risk. Incorporating evolutionary parameters for bacterial pathogens has been a challenge, particularly due to the complexity created by horizontal gene transfer in multi-strain species, leaving model outputs with a high degree of uncertainty. Recent models attempt to take advantage of the detailed evolutionary knowledge provided by availability of longitudinal population genomics datasets. Models based on the balancing of individual gene frequencies across a pathogen species population, termed ‘negative frequency-dependent selection’, have been applied to provide plausible, high-resolution explanations for population responses to vaccines [77] and emergence of pathogenic strains [109]. This approach has also been applied to hypothesise PCV formulations that could be tailored to the extant population and provide better disease prevention [110]. A key strength of this approach is that it could allow for region-specific vaccine design, addressing the reality that pathogen populations can vary significantly across the world and that ‘one size fits all’ global vaccines may not be the optimum approach. The WHO also lists a number of ‘pipeline’ vaccines and many others are in early design stages. Population genomics is increasingly prioritised in vaccine design and is further employed as the foundation of other powerful ‘omics’ approaches, such as surveying potential immunogenicity across complete proteome arrays [111].

### Potential application of genomics in clinical microbiology laboratories

Genomic technologies have the potential to provide solutions for the inherent challenge of isolating the pathogen in cases of disease. Failure to culture the live pathogen from a clinical sample is not uncommon and molecular techniques are being developed that aim to extract and analyse the pathogen DNA directly rather than relying on the presence of viable pathogen cells. If these techniques can be honed to enrich whole genomes, then clinical pathogen genomic protocols for some species could become ‘culture-free’. Another potential benefit of genomics comes from the correlation and derivation of important pathogen phenotypes that are normally determined through an array of wet-lab techniques, often with species-specific protocols and each requiring maintenance of lab infrastructure and spend on consumables. A number of studies have shown a high degree of concordance for deriving such phenotypes directly from genomic data and many public health labs are choosing genomics as their main, or only method for their determination [112, 113].

In conclusion, overcoming the above challenges requires multi-disciplinary expertise, support from the government and sufficient funding. The approach taken and lessons learned from the GPS project discussed in this review—surveillance priority and infrastructure, collaboration models, portfolio of capacity building and bioinformatics training, solutions to challenges in LMICs, recent advances in genomics—may guide generic surveillance networks at national and international level.

## Data Availability

Data sharing is not applicable to this article as no datasets were generated or analysed during the current study.

## Abbreviations

PCV: Pneumococcal conjugate vaccine
LMICs: Low- and middle-income countries
AMC: Advance Market Commitment
IPD: Invasive pneumococcal disease
WGS: Whole-genome sequencing
CPS: Capsular polysaccharide
PPVs: Pneumococcal polysaccharide vaccines
VT: Vaccine serotype
NVT: Non-vaccine serotype
